# If You Don't Have Valence, Ask Your Neighbor: Evaluation of Neutral Words as a Function of Affective Semantic Associates

**DOI:** 10.3389/fpsyg.2017.00343

**Published:** 2017-03-13

**Authors:** Michael Kuhlmann, Markus J. Hofmann, Arthur M. Jacobs

**Affiliations:** ^1^Department of Education and Psychology, Free University BerlinBerlin, Germany; ^2^Department of Psychology, University WuppertalWuppertal, Germany; ^3^Department of Education and Psychology, Dahlem Institute for Neuroimaging of Emotion, Free University BerlinBerlin, Germany; ^4^Department of Education and Psychology, Center for Cognitive Neuroscience, Free University BerlinBerlin, Germany

**Keywords:** neutral words, valence, ambivalence, semantic processing, co-occurrence networks

## Abstract

How do humans perform difficult forced-choice evaluations, e.g., of words that have been previously rated as being neutral? Here we tested the hypothesis that in this case, the valence of semantic associates is of significant influence. From corpus based co-occurrence statistics as a measure of association strength we computed individual neighborhoods for single neutral words comprised of the 10 words with the largest association strength. We then selected neutral words according to the valence of the associated words included in the neighborhoods, which were either mostly positive, mostly negative, mostly neutral or mixed positive and negative, and tested them using a valence decision task (VDT). The data showed that the valence of semantic neighbors can predict valence judgments to neutral words. However, all but the positive neighborhood items revealed a high tendency to elicit negative responses. For the positive and negative neighborhood categories responses congruent with the neighborhood's valence were faster than incongruent responses. We interpret this effect as a semantic network process that supports the evaluation of neutral words by assessing the valence of the associative semantic neighborhood. In this perspective, valence is considered a semantic super-feature, at least partially represented in associative activation patterns of semantic networks.

## Introduction

“I have some good news and some bad news.” This common introduction invites to an affective round-trip. The words “good” and “bad,” verbal stimuli with positive and negative valence, inform about the valence of the entire announcement. In everyday life, the quasi incessant and often unconscious evaluation of stimulus valence provides us with critical information for making decisions and choosing actions that are situation-adequate (Lebrecht et al., 2012). The concept of valence is an integral part of many theories of emotion claiming that the multitude of emotional experiences like states of anger, fear, disgust, or happiness are derived from a core affect that is composed of valence and a second major dimension, representing the general grade of emotional activation, called arousal (e.g., Wundt, 1896; Osgood et al., 1957; Russell, 1980). However, despite its ubiquitous use, valence is not a notion beyond dispute and it remains unclear how, when, and where the brain computes valence signals in even the simplest task, i.e., the valence decision task (VDT) where participants decide whether a stimulus is positive or negative (Maddock et al., 2003; Võ et al., 2006; Jacobs et al., 2015). Recent research therefore focuses on valence as an integral component of mental object representations and on the mechanisms underlying the brain's computation of affective valence from perceptual or semantic representations (e.g., Lebrecht et al., 2012). Assuming that lexico-semantic representations are the result of learning the statistical structure underlying a single joint distribution of both experiential and distributional data (Andrews et al., 2009), valence can be construed as a semantic *super-feature* (Jacobs et al., 2015).

The experiential aspect of the semantic super-feature of valence is gained by extralinguistic, sensory-motor experience with the word's referents. This can be a physical object or an event, thus the experience includes physical features like color and shape, but also pleasantness. Niedenthal (2007) and Niedenthal et al. (2009) elaborate on the relation of the sensory motor system and emotional processing in their theory of embodied emotions.

The distributional aspect, on the other hand, is grounded in the intralingual dependent distribution of words. Texts are usually used to convey meaningful information, and that does not only influence which words to use, but also creates contextual word patterns within a language. Analyzing the distributive word patterns in texts has become a distinct field in computational linguistics. Some of the models produced in this field are well-known in psychology, for instance latent semantic analysis (Landauer and Dumais, 1997), or Bayesian topics models (Griffiths et al., 2007). The dependent distribution of words can be assessed from a large text corpus that is representative for a language by extracting how often words are occurring close to other words, e.g., within the same sentence. Words that are often co-occurring can be considered to be semantically associated (cf. Evert, 2005). In turn, it can be expected that the co-occurrence of words contributes to define their meaning by shaping the neural connection patterns in semantic networks through Hebbian learning style mechanisms (Hebb, 1949; Rapp, 2002). Therefore, co-occurrence enables to model the spread of activation within semantic networks and hence to predict, which words will receive co-activation from the activation of other words (cf. Hofmann and Jacobs, 2014). Empirical evidence that co-occurrence can partially predict the valence of words comes from Westbury et al. (2015). In a recent study they showed that valence ratings of words can be predicted by their co-occurrence based associations to a selected set of emotion labels, derived from theories of basic emotions (cf. also Hofmann and Jacobs, 2014).

A further step should be to disentangle the contribution of experiential and distributional data in the course of the evaluation process. However, the typical very positive and very negative emotion words used in studies on the processing of valence (e.g., Kissler and Herbert, 2013) will preclude to contrast the two types of data. Instead, we propose that this is possible with “neutral” words. To our knowledge, so far, there is yet no theory of emotion really elaborating on the structures and/or processes underlying stimulus neutrality. Since valence typically is conceived as a bipolar continuum, neutrality initially seems to be regarded as a state of no or insignificant valence. Alternatively, the evaluative space model incorporates the possibility of a combination of positive and negative valence for the same stimulus, i.e., mixed emotions (e.g., Norris et al., 2010; Briesemeister et al., 2012). In this prequantitative model stimulus neutrality can theoretically result from a balanced state of positive and negative affect, but the model does not allow to predict for which stimuli this would be the case. According to recent descriptive models of performance in the VDT (Jacobs et al., 2015), stimulus neutrality could result from a balance between distributional and experiential data with, e.g., positive distributional features counterbalanced by negative experiential ones or vice versa. Another possibility is that experiential and distributional features are both truly neutral, i.e., lack any substantial valence information. Again, however, these prequantitative models allow no specific predictions with regard to individual stimuli. On the other hand, computational models of lexical semantics, such as the Associative Read-Out model (Hofmann et al., 2011; Hofmann and Jacobs, 2014), allow to calculate an estimate of the distributional parts of the valence of single words, and thus specify their *neutrality* in more detail. Since these models implement an associative spreading of activation within semantic networks, the neutrality of a given word could also stem from a balance between its positive and negative semantic associates together with a neutral experiential feature.

In the present study, we tested the influence of semantic associates on affective word evaluation in a VDT. The semantic associates were computed beforehand from corpus based co-occurrence statistics. We assumed that the valence of the semantic associates provides a useful quantitative estimate of the distributional properties co-determining the overall valence of the neutral words that were presented as items in our experiment. The associated words conversely were not presented to the participants, but we predicted that spread of activation from reading the target words alone will co-activate their a priori determined associates within the semantic networks of the participants. We hypothesized that response type and times in the VDT using neutral words would be a function of their associates' valence values. In particular, we assumed that items with either a majority of positive or negative associates would receive more responses corresponding to their associates' valence, compared to the “baseline” response type distribution for items whose associates do not tend to positivity or negativity. If the evaluation of the valence of these items is consistent with the valence of their associates, we further expected responses to be sped up and also to be faster compared to the same types of response for items with no tendency to positivity or either negativity in the valence of their associates. Our controls, the items whose associates neither generally tended to positivity nor negativity, were subdivided into items with an even distribution of positive and negative associates and those whose associates had negligibly low valence values. In other words the associates were either an ambivalent mix or in the other case considered as neutral themselves. We selected these two types of control conditions, because we assumed them to be a challenge to evaluate for distinct reasons. The ambivalent condition causes competition of associates, while the neutral condition affords a more thorough search for valence.

## Materials and methods

### Participants

The 19 participants (11 male; aged 19–28; mean 23.5) who took part in our study were right handed, had normal or corrected-to-normal vision and were native speakers of German. They were recruited at the Free University Berlin and gave written informed consent. They either received course credit or were paid for their participation. The study was approved by the ethics committee of the Free University Berlin.

### Materials

We selected our items and associates from words of the BAWL-R (Võ et al., 2006, 2009). Association strength was computed from the German corpus of the “Wortschatz” project (Quasthoff et al., 2006; Hofmann et al., 2011). In general, it is based on the log-likelihood ratio of the actual co-occurrence of two words in a sentence divided by the likelihood expected from the single-word frequencies (Dunning, 1993). For each word of the BAWL-R, we computed the association strength to each other word in the BAWL-R by log-10 transforming the resulting chi-square value. This procedure results in a vector for each word comprised of the association strength values to each other BAWL-R word and ranks the words according to the strength of the association depicted by the chi-square value. The magnitude of association strength values and there distribution is heterogeneous for different words. For example the highest ranking word to one word might have a much larger chi-square value than the highest ranking of another word. Since the role of the magnitude of association strength in cognitive processing is still poorly understood, we resorted only to rank. The highest ranking associates of a given word should predominantly be co-activated by spread of activation. Therefore, and also to minimize computational load, we focused on the 10 highest ranking words by association strength to each word individually, which we will further refer to as semantic neighborhood. We defined words as neutral when their BAWL-R valence values (7 point rating scale from –3 to 3) were between –1 and 1. For these words we calculated mean and sd of valence and arousal of their semantic neighborhood derived from BAWL-R valence and arousal values of the respective neighborhood words. The mean and standard deviation of the valence values of neighborhood words defined the experimental category of the neutral target words. Words with a neighborhood valence sd below 1 were assigned to the positive category when the mean neighborhood valence was larger than 0.8, to the negative category when the mean neighborhood valence was below –0.8, and to the neutral neighborhood category when the mean was between –0.2 and 0.2. When neighborhood valence sd was larger than 1 and the mean was between –0.2 and 0.2 the word was assigned to the ambivalent category. An example of each category together with its neighborhood can be found in Table 1. We selected 50 words from each of the four categories to build an item set with no significant differences in valence, arousal, and imageability mean and sd, and also letter count, syllable count, and word frequency (*t*'s < 1; Baayen et al., 1993, see Table 2). The complete item set is included in Table 3.

**Table 1:** **Example words for each condition with corresponding neighborhood**.

**Condition**	**Positive neighborhood**	**Negative neighborhood**	**Ambivalent neighborhood**	**Neutral neighborhood**
**Word**	**Gelaunt (humored)**	**Justiz (judiciary)**	**Eile (hurry)**	**Gutachten (survey)**
Neighborhood	Entspannt (relaxed)	Untreue (unfaithfulness)	Vorsicht (caution)	Auftrag (assignment)
	Jovial (jovial)	Betrug (fraud)	Sorgfalt (thoroughness)	Entwurf (draft)
	Vergnügt (cheery)	Beihilfe (subsidy)	Sorge (worry)	Bericht (report)
	Locker (casual)	Anklage (prosecution)	Euphorie (euphoria)	Befund (findings)
	Selbstbewusst (self-confident)	Erpressung (blackmail)	Optimismus (optimism)	Aussage (statement)
	Fröhlich (merry)	Staatsanwalt (public prosecutor)	Not (hardship)	Psychiater (psychiatrist)
	Amüsiert (amused)	Kinderschänder (child abuser)	Härte (hardness)	Prüfer (inspector)
	Warmherzig (warm-hearted)	Mord (murder)	Ehrgeiz (ambition)	Ministerium (ministry)
	Ungezwungen (casual)	Meineid (perjury)	Bedeutung (meaning)	Lupe (lens)
	Beschwingt (elated)	Beleidigung (insult)	Panik (panic)	Ergeben (yield)

**Table 2:** **Means of neighborhood and word properties for the experimental conditions with sd in parentheses**.

**Conditions**	**Neighborhood**		**Word**						
	**Valence**	**Arousal**	**Valence**	**Arousal**	**Imageability**	**Frequency**	**#Letters**	**#Syllables**
Positive neighborhood	1.05 (0.22)	2.72 (0.45)	−0.23 (0.39)	2.83 (0.58)	3.86 (1.32)	1.69 (0.84)	6.48 (1.47)	2.42 (0.61)
Negative neighborhood	−1.17 (0.34)	3.31 (0.33)	−0.35 (0.43)	2.98 (0.54)	3.9 (1.26)	1.94 (0.64)	6.6 (1.4)	2.28 (0.7)
Ambivalent neighborhood	−0.01 (0.11)	2.87 (0.29)	−0.33 (0.31)	2.97 (0.38)	3.94 (1.12)	1.93 (0.71)	6.5 (1.43)	2.32 (0.62)
Neutral neighborhood	0.01 (0.11)	2.13 (0.81)	−0.29 (0.37)	2.9 (0.37)	3.85 (1)	1.78 (0.64)	6.76 (1.73)	2.32 (0.65)

**Table 3:** **List of items**.

**Positive**	**Negative**	**Ambivalent**	**Neutral**
ABBILD	ABWESEND	ABKEHR	ABWEHR
ABORDNUNG	AFFEKT	ADEL	ABWURF
ABREISE	ANKLÄGER	AMPEL	AMPULLE
ACHTUNG	ANZEIGE	AMTLICH	AUFOPFERN
ADER	AUSBRUCH	ANZAHLUNG	AUSREIßEN
AKRIBISCH	AUSWURF	APOSTEL	BARACKE
BEGIERDE	BEDENKEN	AUFZUCHT	BARRIKADE
BÖRSE	BEIHILFE	BEFUND	BEENDEN
BÜRO	DESERTEUR	BEICHTE	BESCHLUSS
DISZIPLIN	DETEKTIV	BEKÄMPFEN	BOCK
ELFENBEIN	DISPUT	BENZIN	BROCKEN
ESSAY	ELITÄR	BESETZEN	DATEI
ESSIG	ERHEBEN	BEWERBER	DAUER
FRÜH	EROBERUNG	BEZAHLEN	DELLE
GARDINE	ERSCHÖPFT	DARLEHEN	DICHT
GEKICHER	FILTER	DIAGNOSE	DRÜCKEN
GELÄCHTER	FLUT	DOMINANZ	FLEISCHER
GELAUNT	GEHILFE	DUELL	GEGENSATZ
HERRGOTT	GITTER	EILE	GEGENTEIL
HERRIN	HAUFEN	EREMIT	GURU
HYMNE	HINDERNIS	GESÄß	GUTACHTEN
JOVIAL	HUNGER	HORMON	HÄRTE
KOITUS	IRREN	HYPNOSE	HITZKOPF
KOMITEE	JUSTIZ	INDUSTRIE	KALORIE
LEKTION	KAMMER	INFORMANT	KLINGEL
LISTIG	KAPLAN	INSEKT	LAIE
LITANEI	KOMMUNIST	KÄMPFEN	LAKAI
MATERIELL	KRUMM	KEHLE	LIZENZ
MORAL	MINDER	LANZE	MINIMAL
NACHBAR	MINE	LOSUNG	NOTAR
NEUTRAL	MÖRSER	MASSIV	ÖLIG
NORM	MOTIV	MAUER	PEGEL
ONANIE	OBSZÖN	MILIEU	POKER
ORGIE	PLATT	NEBEL	RAMPE
PASTE	RABIAT	NIERE	RELATION
PHRASE	REUE	PENSUM	RITZE
PLAGIAT	REUIG	PILLE	SCHLEPPEN
REDSELIG	REVISION	PREDIGT	SCHLIEßEN
ROBOTER	SCHARF	PULVER	SELTEN
SEHNEN	SCHIELEN	RAUCH	SPESEN
SITUIERT	SCHLÄFE	REGIEREN	SPUK
TATZE	SEXUELL	RELIGIÖS	TÜMPEL
TOILETTE	SPION	RUCK	ÜBERFLUSS
TÜCKE	STEIF	SKEPSIS	VEREITELN
ÜBUNG	SUBJEKTIV	TROTZEN	VERKEHR
UNKRAUT	TRIBUNAL	UMBRUCH	VOLLMACHT
WAGNIS	VERDACHT	UMZUG	WEGZIEHEN
WINDEL	VORFALL	VERSETZEN	WILDFANG
WODKA	ZAHLUNG	WARTEN	ZERLEGEN
ZEUGNIS	ZEUGE	WINZIG	ZUFÄLLIG

### Procedure

The participants were informed that they could resign their participation at any time without the need of justification or any negative consequences. They then received the instructions on the screen. Their task was to decide whether a word presented for a brief time was either positive or negative and to press one of two buttons accordingly. The assignment of the response buttons was counterbalanced across participants. Participants were told that they would have the possibility to practice the task and to respond within the time window of presentation. They then worked through 10 practice trials and after a short break through the 200 main trials with a short break after half of the trials. Each trial started with a fixation cross in the screen center with a jittered duration between 2,500 and 5,000 ms. The trial continued with the stimulus item being presented for 2,000 ms. The order of item presentation was fully randomized. We collected response of the first button press within item presentation and reaction time (RT). The duration of breaks was left to the decision of the participants. On average they lasted 1 min.

### Analyses

Trials without response were excluded from the analyses (6.5%, *n* = 247). We tested whether the response patterns for each condition were different from chance (0.5 response probability) with χ^2^ tests. Using a nominal-logistic regression we tested experimental condition (positive, negative, neutral, ambivalent) as a predictor for response type (positive, negative). Planed pairwise comparisons tested the conditions with unambiguous, i.e., positive and negative, neighborhoods separately against the ambiguous neighborhood conditions: ambivalent and neutral.

RT data were analyzed with a mixed fixed and random effects model using the Statistical software JMP 11Pro (SAS Institute Inc.). The conditions (positive, negative, neutral, ambivalent) and response type (positive, negative) nested into participants were modeled as a fixed effect. Although we had controlled variables that are known to affect latencies in the processing of words, we also inserted word valence, word arousal, word imageability, word frequency, number of letters, and number of syllables as covariates to achieve a more detailed model of data variance. For the same reason we also inserted mean neighborhood arousal as a covariate. Participants and items nested within conditions were modeled as random effects.

## Results

### Responses

There was a shift of the response ratio. Positive neighborhood items had more positive than negative responses. The neutral and ambivalent neighborhood items had more negative than positive responses at a similar level. The negative neighborhood items had more negative than positive responses to even a larger extent. The responses to each single condition were significantly different from a chance-distribution. There was a significant effect of experimental condition on the response type [$\chi_{\left(3,\;\text{N}=3553\right)}^{2}$ = 94.32, *p* < 0.001, Nagelkerkes *R*^2^ = 0.04]. Planned comparisons revealed that positive neighborhood items were significantly different from ambivalent neighborhood items [$\chi_{\left(1,\;\text{N}=1777\right)}^{2}$ = 44.56, *p* < 0.001, odds ratio = 0.54] and from neutral neighborhood items [$\chi_{\left(1,\;\text{N}=1769\right)}^{2}$ = 29.73, *p* < 0.001, odds ratio = 0.59]. Likewise, negative neighborhood items were significantly different from ambivalent [χ^2^_(1, *N* = 1784)_ = 7.78, *p* = 0.005, odds ratio = 1.31] and neutral [χ^2^_(1, *N* = 1776)_ = 16, *p* < 0.001, odds ratio = 1.48] neighborhood items. These effects are based on a shift of the response ratio from (i) more positive than negative responses for positive neighborhood items, to increasingly more negative than positive responses in the order of (ii) neutral, (iii) ambivalent, and maximally for (iv) negative neighborhood items (see Table 4).

**Table 4:** **χ^**2**^ tests vs. 0.5 probability with 95% CI**.

**Condition**	**χ^2^ (1)**	***N***	***p***	**Positive response**			**Negative response**		
				**Prob**	**Lower CI**	**Upper CI**	**Prob**	**Lower CI**	**Upper CI**
Positive	15	885	<0.001	0.56	0.53	0.6	0.44	0.4	0.47
Negative	89.4	892	<0.001	0.34	0.31	0.37	0.66	0.63	0.69
Ambivalent	31.07	892	<0.001	0.41	0.38	0.44	0.59	0.56	0.62
Neutral	14.74	884	<0.001	0.44	0.4	0.47	0.56	0.53	0.6

### Reaction times

**Figure 1:**
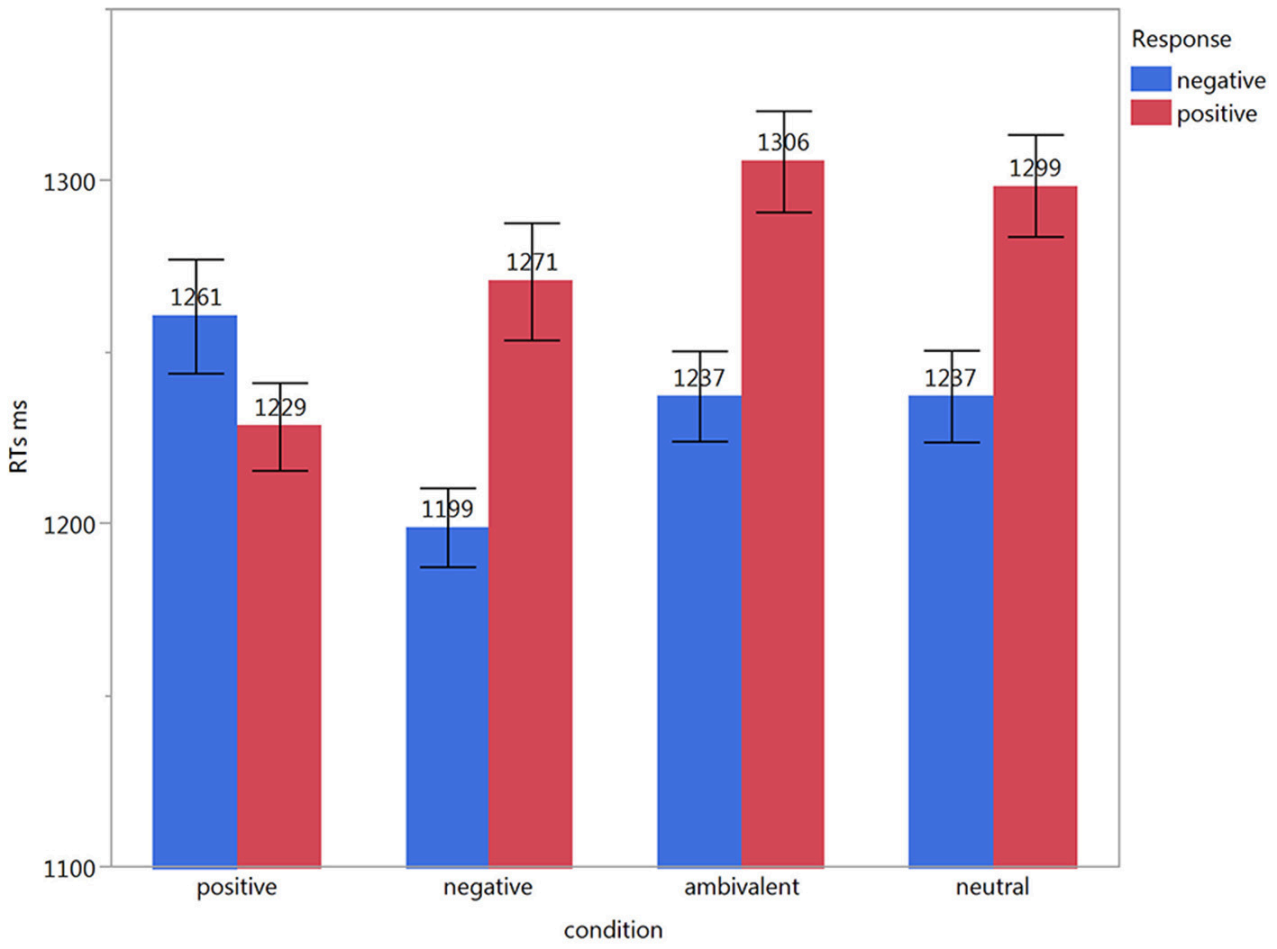
**Mean RTs for responses given in each condition**. Error-bars represent standard error.

For RTs, the main effects of condition (positive, negative, ambivalent, neutral) [*F*_(3, 181)_ = 1.93, *p* = 0.13] and response [positive, negative; *F*_(1, 3217.8)_ = 2.69, *p* = 0.1] were not significant. However, we found a significant effect for the interaction between condition and response type [*F*_(3, 3088.3)_ = 3.87, *p* = 0.01]. Pairwise comparisons revealed no significant effects. Descriptively they showed the following differences: Considering condition alone, negative neighborhood items produced the fastest responses shortly followed by positive neighborhood items. Neutral and ambivalent neighborhood items were considerably slower. When taking the given response into account, responses to negative and positive neighborhood items that were congruent with the respective neighborhood valence were faster than incongruent responses. Neutral and ambivalent neighborhood items had similar latencies with generally faster negative responses than positive ones (see Figure 1). The covariates valence, arousal, word frequency, number of letters, and number of syllables revealed no significant effects, while imageability revealed a significant effect [*F*_(1, 174)_ = 3.99, *p* = 0.05].

## Discussion

The influence of neighborhood valence was apparent in the pattern of responses in the present VDT. Although all items were neutral as established by previous valence ratings, positive neighborhood items elicited more positive responses and negative neighborhood items produced more negative responses than items with a neutral neighborhood. This suggests that a more or less tacitly retrieved positive or negative language context co-determines the valence of a given word (Harris, 1951).

While there is extensive co-occurrence data, the more limited amount of available valence data prevents from applying our computational procedure to any word. Moreover, it limits the pool of associates for the semantic neighborhoods. Still our results show that they were sufficient for estimating the distributive aspect of valence. This gives rise to the assumption that the distribution of valence in associates without available valence ratings does not crucially deviate.

We also found that ambivalent and neutral neighborhood items showed a negativity bias with more negative responses than expected by chance. This is consistent with recent data obtained in the VDT. When noun-noun compounds are composed of both, a negative and a positive word, participants judge them to be relatively negative (Jacobs et al., 2015). A dominance of negativity over positivity in emotion is often found (see Baumeister et al., 2001). Rozin and Royzman (2001) stated that evaluations tend to be more negative than the algebraic sum of integrated positive and negative information would predict and Ito et al. (1998) presented evidence that the negativity bias originates at the stage of evaluative categorization. Moreover, such a negativity bias is also well known in many other tasks, when a great amount of affective information is available (Norris et al., 2010). Norris et al. (2010, p. 431) suggested “that under conditions in which little to no affective information is available…, positivity outweighs negativity.” Thus, the present negativity bias suggests that associations in semantic networks can bring a significant amount of valence information into the evaluative space of actually neutral words, although the affective information is generated by an internal process and not triggered by additional external stimuli. This dominance of affective contextual word features was also present in the RT data. Thus, items with an unequivocal positive or negative semantic neighborhood were evaluated faster than those with an ambivalent or neutral neighborhood. Moreover, for items with ambivalent and neutral semantic neighborhoods, we found that negative responses were faster than positive responses. Thus, much as our recently observed faster RTs in ambivalent, directly available valences of noun–noun compounds consisting of a positive and negative word (cf. Jacobs et al., 2015; Kuhlmann et al., 2016), a negativity bias can also be elicited by absent, but associated words. This finding corroborates the notion that a large amount of affective information can spread from affective words to its directly associated neutral neighbors, which can also be used to predict the valence of a word (Recchia and Louwerse, 2014).

In sum, our results can be explained in terms of spreading (associative) activation models. For example Bower (1981) proposed that positive or negative valence can be considered a node in a semantic network (cf. Schröder and Thagard, 2013). Such a positive and negative “super-feature unit” could be added to computational models accounting for orthographic, phonological, or semantic neighborhood effects (Grainger and Jacobs, 1996; Jacobs et al., 1998; Hofmann et al., 2011; Hofmann and Jacobs, 2014) to allow judgments of the valence of a word. Thus, if no valence information is available for a stimulus, associated items become co-activated (Collins and Loftus, 1975; Hofmann and Jacobs, 2014), and thus the meaning of these items co-resonates (Hofmann et al., 2011; Baayen et al., 2016), the resonance spreading toward super-feature units finally determining word valence (Hofmann et al., 2011).

If a great amount of associated word units activate the negative unit, a “negative” response is given, and vice versa for positive words. If the valence of most of the neighbors spreads toward either the positive *or* the negative super-feature units, more evidence is fed forward within the same amount of time (cf. Grainger and Jacobs, 1996), and thus responses are faster than in neutral or ambivalent neighborhoods. If there is an associative spread toward positive *and* negative super-feature units, this leads to competition (Botvinick et al., 2001), and thus RTs are delayed. Similarly, responses are delayed, when activation must spread across several intermediate neutral units, to reach the criterion level sufficient to execute a (binary) valence response. Thus, it takes you more time to know the valence of a word by the positive or negative company it kept during its learning history (cf. Firth, 1957).

## Ethics statement

Ethikkommission der Freien Universität Berlin Free University Berlin Habelschwerdter Allee 14195 Berlin Before the experiment all participants were verbally informed that they could resign from participation at any time without explanation and without negative consequences. No vulnerable populations were involved

## Author contributions

MK conducted the analyses and prepared figures and tables. MK, MH, and AJ wrote the manuscript.

## Funding

This research was partly supported by grants from the Deutsche Forschungsgemeinschaft to AJ (JA823/4-2) and to MH (HO5139/2-1).

### Conflict of interest statement

The authors declare that the research was conducted in the absence of any commercial or financial relationships that could be construed as a potential conflict of interest.
